# Contrasting Effects of Environmental Concentrations of Sulfonamides on Microbial Heterotrophic Activities in Freshwater Sediments

**DOI:** 10.3389/fmicb.2021.753647

**Published:** 2021-11-03

**Authors:** Stéphane Pesce, Laura Kergoat, Laurianne Paris, Loren Billet, Pascale Besse-Hoggan, Chloé Bonnineau

**Affiliations:** ^1^Université Clermont Auvergne, CNRS, Institut de Chimie de Clermont-Ferrand, Clermont-Ferrand, France; ^2^Université Clermont Auvergne, CNRS, Sigma Clermont, Institut de Chimie de Clermont-Ferrand, Clermont-Ferrand, France; ^3^AgroSup Dijon, INRAE, Université de Bourgogne Franche-Comté, Agroécologie, Dijon, France

**Keywords:** β-glucosidase, benthic, biogeochemical cycles, microbial ecotoxicology, respiration, sulfamethazine, sulfamethoxazole, sorption

## Abstract

The sulfonamide antibiotics sulfamethoxazole (SMX) and sulfamethazine (SMZ) are regularly detected in surface sediments of contaminated hydrosystems, with maximum concentrations that can reach tens of μg kg^–1^ in stream and river sediments. Little is known about the resulting effects on the exposed benthic organisms. Here we investigated the functional response of stream sediment microbial communities exposed for 4 weeks to two levels of environmentally relevant concentrations of SMX and SMZ, tested individually. To this end, we developed a laboratory channel experiment where natural stream sediments were immersed in water contaminated with nominal environmental concentrations of 500 and 5,000 ng L^–1^ of SMX or SMZ, causing their accumulation in surface sediments. The mean maximum concentrations measured in the sediment (about 2.1 μg SMX kg^–1^ dw and 4.5 μg SMZ kg^–1^ dw) were consistent with those reported in contaminated rivers. The resulting chronic exposure had various effects on the functional potential of the sediment microbial communities, according to the substance (SMX or SMZ), the type of treatment (high or low) and the measured activity, with a strong influence of temporal dynamics. Whereas the SMZ treatments resulted in only transient effects on the five microbial activities investigated, we observed a significant stimulation of the β-glucosidase activity over the 28 days in the communities exposed to the high concentration of SMX. Together with the stimulation of aerobic respiration at low SMX concentrations and the reduced concentration observed in the last days, our results suggest a potential biodegradation of sulfonamides by microbial communities from sediments. Given the key functional role of surface sediment microbial communities in streams and rivers, our findings suggest that the frequently reported contamination of sediments by sulfonamides is likely to affect biogeochemical cycles, with possible impact on ecosystem functioning.

## Introduction

Among contaminants of emerging concern, antibiotics are increasingly detected in aquatic ecosystems worldwide, reflecting their widespread use (Chen and Zhou, 2014; Kairigo et al., 2020; Bonnineau et al., 2021; Zheng et al., 2021). Case reports and reviews on the contamination of these ecosystems by antibiotics show that sulfonamides, especially sulfamethoxazole (SMX) and sulfamethazine (SMZ), are among the most frequently detected substances (e.g., Kovalakova et al., 2020; Zheng et al., 2021). Owing to the frequent occurrence of SMZ and SMX in surface waters, there has been growing interest in assessing their toxicity toward various aquatic organisms. These include model species, such as the cyanobacterium *Synechococcus leopoliensis*, the protist *Tetrahymena pyriformis*, the microalga *Pseudokirchneriella subcapitata* and the crustacean *Daphnia magna*, with reported NOEC (no observed effect concentration) ranging from several μg L^–1^ to several mg L^–1^ (Yang et al., 2008; De Liguoro et al., 2009; Straub, 2016; U.S. Environmental Protection Agency, 2019). However, the suitability of ecotoxicological tests at species or population level to assess the ecological effects of antibiotics is controversial (Boxall et al., 2012; Brandt et al., 2015). In particular, microbial communities are rarely taken into account in these types of test (Brandt et al., 2015), even though they are key players in a broad range of ecological processes that are essential to ecosystem functioning and that contribute to ecosystem services (Cavicchioli et al., 2019; Pesce et al., 2020).

Only a few studies have investigated the effects of SMZ and SMX on aquatic microbial communities (Grenni et al., 2018). Most have focused on periphytic biofilms, which are complex assemblages that develop on inert substrata, such as pebbles or cobbles (Yergeau et al., 2010, 2012; Johansson et al., 2014; Kergoat et al., 2021). Using Biolog Ecoplates, Johansson et al. (2014) estimated that the effective concentrations (CE) of SMX inhibiting 10 and 50% of total carbon source utilization by marine biofilms after 4 days of exposure were respectively, close to 14 μg L^–1^ (CE_10_) and 272 μg L^–1^ (CE_50_). Yergeau et al. (2010, 2012) showed that more environmentally relevant concentrations of SMZ or SMX (500 ng L^–1^ of either) could affect community composition and the meta-transcriptome of river biofilms exposed for 8 weeks. More recently, Kergoat et al. (2021) also observed changes in the bacterial and diatom composition of river biofilms exposed for 4 weeks to 500 and 5,000 ng L^–1^ of SMX or SMZ (tested individually at these two nominal concentrations). Moreover, they observed slight and transitory effects on microbial heterotrophic functions such as the phosphatase and leucine aminopeptidase enzymatic activities (Kergoat et al., 2021). Paumelle et al. (2021) also showed slight functional effects of SMX and SMZ on microbial decomposers associated with leaf litter. They reported a significant increase in the β-glucosidase activities of these communities throughout 16 days exposure to a nominal concentration of 5,000 ng L^–1^ of SMX or SMZ, tested individually. However, no effect was observed on the other enzymatic activities they measured (cellobiohydrolase, phenol oxidase, alkaline phosphatase, and leucine aminopeptidase) or on the decomposition rate of the colonized leaves (Paumelle et al., 2021). Interestingly, the above cited studies are evidence that the microbial response to chronic exposure to SMX and SMZ can differ according to the substance, despite their similar molecular structures and modes of action (Yergeau et al., 2010, 2012; Kergoat et al., 2021; Paumelle et al., 2021).

Besides their frequent occurrence in surface waters, SMX and SMZ are also regularly detected in surface sediments of contaminated hydrosystems (Chen and Zhou, 2014; Shi et al., 2014; De Vries and Zhang, 2016; Bonnineau et al., 2021; Zheng et al., 2021). Experimental studies have demonstrated that the sorption rates of these antibiotics, which vary among sediments and antibiotics in water-sediment systems, can be very high (Carstens et al., 2013; Li and Cui, 2020). In their recent systematic review of antibiotics in surface sediments of estuarine and coastal environments, Zheng et al. (2021) reported mean concentrations of SMX and SMZ close to 9 and 2 μg kg^–1^ respectively, with maximum values reaching about 100 μg kg^–1^ for SMX and 20 μg kg^–1^ for SMZ. In freshwater ecosystems, De Vries and Zhang (2016) compiled the results of 11 studies assessing the occurrence of antibiotics in stream and river sediments mainly located in high-intensity agricultural regions and subjected to wastewater discharge and agricultural runoff. The reported median and maximum concentrations were respectively, 0.52 and 7.86 μg kg^–1^ for SMX and 2.87 and 248 μg kg^–1^ for SMZ (De Vries and Zhang, 2016, and references therein). In a case study conducted in the Eastern province of Kenya, Kairigo et al. (2020) observed maximum concentrations of SMX of about 900 μg kg^–1^ in surface sediments located downstream of the Machakos town wastewater treatment plant.

Despite very few studies on the effects of SMX and SMZ on sediment microbial communities, it has been evidenced that the contamination of sediments by this type of substance can impact microbial processes involved in the N cycle (Hou et al., 2015; Xu et al., 2020; Chen et al., 2021). In a sediment slurry incubation experiment with a dose-response design, Hou et al. (2015) described a negative exponential relationship between denitrifying gene abundances and SMZ concentrations. Increasing concentrations of SMZ led to increased depression of denitrification rates (Hou et al., 2015). Similar results were obtained by Xu et al. (2020) and Chen et al. (2021), who exposed river sediments to increasing concentrations of SMX. All these studies showed that the contamination of sediments by high concentrations of SMX or SMZ could increase the emission of nitrous oxide (N_2_O) and alter the ecological functioning of the benthic compartment. However, further research is needed to better assess the potential effects of environmental contamination by SMX and SMZ on sediment functioning. More generally, it is recognized that efforts are needed to improve the ecological risk assessment of freshwater sediments by better assessing the ecotoxicological effects in this compartment at community level (Pesce et al., 2018).

In this context, we investigated the functional response of stream sediment microbial communities exposed for 4 weeks to two levels of environmentally relevant concentrations of SMX and SMZ, tested individually. To this end, we developed a microcosm approach, using laboratory channels where natural stream sediments were immersed in water contaminated with nominal concentrations of 500 and 5,000 ng L^–1^ of SMX or SMZ, chosen to reflect environmental concentrations (Deng et al., 2018). The recirculating water was replenished weekly and the resulting sorption of antibiotics in sediment was assessed by weekly concentration measurements in this compartment. Resulting ecotoxicological effects on the exposed sediment microbial communities were assessed over time by measuring 5 potential microbial activities (the enzymatic activities: β-glucosidase, leucine aminopeptidase, and phosphatase and the metabolic activities: aerobic respiration and denitrification). This panel of activities was chosen to take into consideration the three most important nutrient cycles to which microbial communities contribute, namely the C (β-glucosidase and aerobic respiration), N (leucine aminopeptidase and denitrification), and P (phosphatase) cycles. Moreover, these activities are commonly measured in microbial ecotoxicology (e.g., Tlili et al., 2017; Paumelle et al., 2021) allowing comparison across studies.

## Materials and Methods

### Experimental Design

Natural sediment and associated communities were sampled on February 2018 at the upstream section of the Morcille river (Saint Joseph, 46°10′39.1″N 4°38′10.1″E), located in a forested non-urban area, upstream of vineyards. About 150 kg of wet surface sediment (0–3 cm) was collected using an Ekman grab, sieved at 2 mm, and brought to the laboratory. After sieving, sediment particle-size classes were distributed as follows: 48.8% (500 μm–2 mm), 30.7% (200–500 μm), 12.5% (50–200 μm), 8.0% (<50 μm), corresponding to a relatively coarse sediment, with a low organic matter content (3.1%) and a relatively high water content (36.1%).

The microcosms used for the experiment consisted of 15 glass indoor channels (length×width×height = 83cm×11cm×10cm), as previously described in Mahamoud Ahmed et al. (2018). After sediment homogenization, the bottom of each channel was spread with 7.2 kg wet weight (ww) of sediment and filled with 10 L of circulating water (2:1 v/v demineralized water:groundwater). The experimental design was similar to that used by Kergoat et al. (2021). Briefly, five experimental treatments (with independent triplicate channels) were tested: (i) control condition without SMX or SMZ, (ii) low-SMX: sediment immersed in water contaminated with SMX, at a nominal concentration of 500 ng L^–1^; (iii) high-SMX: sediment immersed in water contaminated with SMX, at a nominal concentration of 5,000 ng L^–1^, (iv) low-SMZ: sediment immersed in water contaminated with SMZ, at a nominal concentration of 500 ng L^–1^, and (v) high-SMZ: sediment immersed in water contaminated with SMZ, at a nominal concentration of 5,000 ng L^–1^. The experiment lasted 4 weeks and water was replenished weekly (for more details of the experimental procedure, see Kergoat et al., 2021).

Sediments were sampled for antibiotic and microbial analyses at the beginning of the experiment (Day 0, D0) and after 7 (D7), 14 (D14), 21 (D21), and 28 (D28) days. At each sampling time (i.e., before water replenishment), about 250 g of sediment was collected uniformly in each channel (using subsamples randomly distributed over the length of the channel), homogenized, and subsampled for further analyses.

### Sediment Chemical Analyses

SMX and SMZ concentrations in the sediment were measured at each sampling time (except for the REF treatment, for which only D0 and D28 samples were analyzed). After sampling, sediments were freeze-dried and crushed; 6.0000 ± 0.0005 g of sediment sample was then added to Nalgene PPCO 50 mL centrifuge tubes (previously checked for lack of SMX and SMZ sorption capacity) together with 50 μL of a 5,000 μg L^–1^ SMX-d4 (Toronto Research Chemicals) and SMZ-d4 (LCG standards) mixture and 20 mL of MeOH/H_2_O 4/1 (v/v) extractant. The tubes were stirred for 18 h using an orbital shaker (Heidolph Reax) at 50 rpm and room temperature. After centrifuging (12,500 × *g* for 15 min), the supernatant was concentrated in a Speed-Vac vacuum concentrator heated at 35°C. The residue was then dissolved in 10 mL of distilled water. After adjusting the pH to 3.8 ± 0.2, samples were concentrated 20-fold on Oasis HLB-500 mg cartridges (WatersTM) according to the manufacturer’s recommendations (elution: 10 mL methanol). The methanol fraction was concentrated in a Speed-Vac vacuum concentrator at room temperature, and the residue was dissolved in 500 μL HPLC-grade water. Antibiotics were assayed by LC/ESI-MS on a Thermo Scientific UHPLC Ultimate 3000 RSLC coupled to an Orbitrap Q-Exactive analyzer. The analyses were carried out in positive mode. The UHPLC was equipped with a Luna Omega Polar C18 column, 100 × 2.1 mm, 1.6 μm (Phenomenex) at 30°C with acetonitrile gradient + 0.1% formic acid (Solvent A) and water + 0.1% formic acid (Solvent B) 0–2.5 min, 30–64.5% A (linear) 2.5–2.6 min, 64.5–99% A (linear) 2.6–5 min, 99% A 5–5.1 min, 99–30% A 5.1–8 min, and 30% A. Flow rate was 0.45 mL min^–1^. For the mass spectrometer, gaseous nitrogen was used as nebulizer gas (50 AU). The spray voltage was 3.0 kV. The limit of quantification for SMX and SMZ was 0.08 μg kg^–1^ of dry sediment.

### Analyses of Sediment Microbial Community Functions

Potential microbial activities for aerobic respiration, denitrification, β-glucosidase, phosphatase, and leucine aminopeptidase were measured using the protocols previously described by Mahamoud Ahmed et al. (2018) and adapted from Foulquier et al. (2013).

Briefly, to measure aerobic respiration and denitrification rates, 10 g of wet sediment was immersed in 10 mL of distilled water under aerobic conditions (aerobic respiration) or 10 mL of a KNO_3_ (2.16 g L^–1^) solution under anaerobic conditions (denitrification) in 150 mL glass flasks. After 2 h and 5 h, headspace gases were sampled and analyzed by gas chromatography on an MTI 200 microcatharometer (MTI Analytical Instruments). Aerobic respiration and denitrification were expressed as ng of CO_2_ or N_2_O per g of sediment dw^–1^ h^–1^, respectively. To measure β-glucosidase, phosphatase, and leucine aminopeptidase enzymatic activities, 1.2 g of wet sediment was incubated for 30 min with pre-determined optimal concentrations of fluorogenic substrates, namely 4-methylumbelliferyl-β-D-glucopyranoside (MUF-Glu, CAS No. 18997-57-4) for β-glucosidase, 4-methylumbelliferyl phosphatase (MUF-P, CAS No. 3368-04-5) for phosphatase, and L-leucine-7-amido-4-methylcoumarin hydrochloride (Leu-AMC, CAS No. 62480-44-8) for leucine aminopeptidase. The activities were stopped with glycine buffer (0.05 M glycine, 0.2 M NH_4_OH, pH 10.4), the samples were centrifuged, and fluorescence was measured (excitation wavelength 360 nm, emission wavelength 460 nm) using a microplate reader (Synergy HT BioTek Instruments). Standard curves of MUF (CAS No. 90-33-5) and AMC (CAS No. 26093-31-2) were plotted to convert fluorescence values into enzymatic activities. Results were then expressed as nmol of hydrolyzed compound per g of sediment dw^–1^ h^–1^.

### Data Analyses

Data (microbial parameters and sulfonamide concentrations) were checked for normality [Shapiro-Wilk test on ANOVA (analysis of variance) residuals, (Royston, 1982)] and homoscedasticity (Fligner-Killeen test, Conover et al., 1981). For each treatment, differences in SMX or SMZ concentrations across time were assessed by a repeated measure ANOVA followed by a pairwise *t*-test with Bonferroni adjustment. Maximal SMX and SMZ concentrations were compared using a *t*-test. For each sampling time, differences in microbial parameters between treatments (low-SMX, high-SMX, low-SMZ, or high-SMZ) and reference were assessed by ANOVA followed by a Dunnett *post hoc* test. To explore correlations between microbial parameters and sulfonamide concentrations in sediments, Pearson correlations were estimated for certain treatments. For all analyses, results were considered as significant if *p* < 0.05. Statistical analyses were performed using R software version 3.4.4 (R Core Team, 2020). Most figures were plotted using R package ggplot2 (Wickham, 2016).

## Results and Discussion

### Sulfamethazine Had a Greater Sediment Sorption Capacity Than Sulfamethoxazole

**FIGURE 1:**
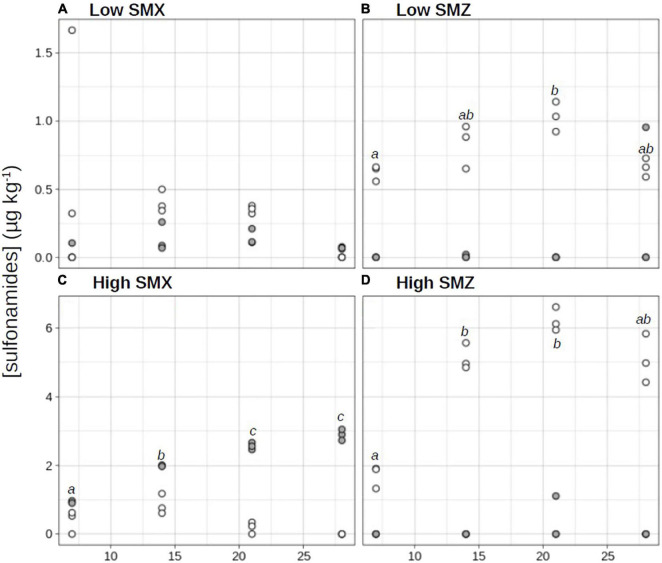
Time-course evolution of sulfamethoxazole (SMX; •) and sulfamethazine (SMZ; ∘) concentrations in sediments exposed to water contaminated by sulfonamides at low **(A,B)** or high **(C,D)** concentrations. Different letters indicate significant differences across time between SMX or SMZ concentrations (repeated measure ANOVA followed by a pairwise *t*-test, *p* < 0.05).

Despite the lack of identified potential sources of contamination by sulfonamides in the sampled river section, a low concentration of SMZ was detected in the collected sediment (0.06 ± 0.10 μg kg^–1^ dw at D0; Figures 1B,D), while no SMX was detected (limit of detection 0.08 μg kg^–1^ dw). The SMZ and SMX analysis performed at D28 in the REF sediment confirmed the lack of SMX contamination and showed that SMZ concentrations in the non-spiked sediment were relatively stable over the experiment (0.10 ± 0.10 μg kg^–1^ dw at D28; data not shown). Unexpectedly, sediments in the SMX treatments also contained low concentrations of SMZ.

The sediment sorption capacity of organic contaminants (such as sulfonamide antibiotics) depends on several variables, including the intrinsic properties of the contaminant and the physical and chemical properties of the water-sediment system (Luo et al., 2019; Huang et al., 2020). In our experimental conditions, SMZ accumulated more in sediment than SMX (Figure 1). The maximum concentrations of SMZ accumulated in sediments were significantly higher than the maximum concentrations of SMX (*t*-test, *p* < 0.05). Indeed in “low” conditions, the maximal concentration in SMZ (1.03 ± 0.11 μg kg^–1^ dw at D21; Figure 1B) was 7-fold higher than in SMX (0.14 ± 0.05 μg kg^–1^ dw at D21; Figure 1A), in “high” conditions, the maximal SMZ concentration (6.20 ± 0.32 μg kg^–1^ dw at D21; Figure 1D) was 2-fold higher than the maximal SMX concentration (2.89 ± 0.16 μg kg^–1^ dw at D28; Figure 1C). This result is in line with those of Zhong et al. (2013), who with two types of lake sediments reported a higher sorption capacity of SMZ than of SMX. However, at both low and high treatments, SMZ concentrations increased exponentially in sediments from D0 to D21 before decreasing from about 18% (high-SMZ, Figure 1D) and 36% (low-SMZ, Figure 1B) between D21 and D28. Two non-exclusive hypotheses can be proposed to explain the observed decrease in the sediment concentration of SMZ at the end of the experiment. The first one is release of the tested contaminant from the sediment to the overlying water (i.e., desorption process; Chen et al., 2014). The second is photodegradation and/or biodegradation processes leading to the dissipation and/or transformation of the studied antibiotics (Carstens et al., 2013; Li and Cui, 2020). These processes could be more pronounced at the end of the experiment owing to the adaptation of some sediment microorganisms after chronic exposure to the tested sulfonamide, which could enhance their ability to co-metabolize and biodegrade this antibiotic (Ricken et al., 2013; Topp et al., 2013; Woappi et al., 2016; Martin-Laurent et al., 2019).

Based on the concentrations measured weekly from D7 to D28, the mean levels of exposure of sediment microbial communities to SMX and SMZ were respectively, 2.10 ± 0.86 μg SMX kg^–1^ dw and 4.53 ± 1.95 SMZ μg kg^–1^ dw in the high treatment and 0.09 ± 0.05 μg SMX kg^–1^ dw and 0.79 ± 0.19 SMZ μg kg^–1^ dw in the low treatment. Based on the existing literature, the high-treatment conditions can be considered as relatively high, but were relevant environmental concentrations, significantly lower than maximum concentrations recently reported in stream and river sediments (8 μg kg^–1^ for SMX and 248 μg kg^–1^ for SMZ; De Vries and Zhang, 2016, and references therein) or estuarine sediments (100 μg kg^–1^ for SMX and 20 μg kg^–1^ for SMZ; Zheng et al., 2021). Similarly, the low-treatment conditions were representative of low environmental concentrations, the measured values being significantly lower than the mean and median values reported in the studies cited above (e.g., 0.52 μg SMX kg^–1^ and 2.87 μg SMZ kg^–1^ in stream and river sediments; De Vries and Zhang, 2016, and references therein).

### Environmental Concentrations of the Tested Sulfonamides Affected Sediment Microbial Community Functions Differently

**FIGURE 2:**
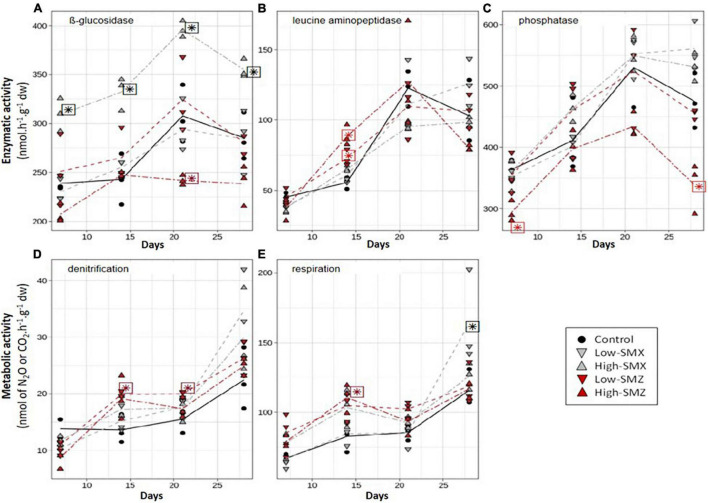
Time course of β-glucosidase **(A)**, leucine aminopeptidase (LAP, **B**), phosphatase **(C)**, denitrification **(D)**, and respiration **(E)** activities in sediments exposed to water contaminated by low or high concentrations of sulfamethoxazole (SMX) and sulfamethazine (SMZ). Stars indicate significant difference from control.

The time course of the functional response of sediment microbial communities to chronic exposure to environmental concentrations of SMX or SMZ was assessed by weekly measurement of five potential activities respectively, contributing to C (β-glucosidase and aerobic respiration), N (leucine-aminopeptidase and denitrification), and P (phosphatase) cycles (Figure 2). Most of the microbial activities increased over time, reflecting microbial growth and activity. As previously reported in several studies assessing the effects of SMX and SMZ on aquatic microbial biofilms (Yergeau et al., 2010, 2012; Kergoat et al., 2021; Paumelle et al., 2021), the functional responses observed in the present experiment varied markedly according to the antibiotic tested.

The SMX treatments significantly affected the two activities involved in the C cycle: β-glucosidase (Figure 2A) and aerobic respiration (Figure 2E). In sediments exposed to the high-SMX treatment, β-glucosidase activity was significantly higher than in the control throughout the experiment (*p* < 0.05 from D7 to D28, Figure 2A) whereas this activity was similar to controls in sediments exposed to low concentrations of SMX. In addition, a positive correlation (Pearson correlation coefficient *r* = 0.733, *p* < 0.05) was found between glucosidase activity and SMX concentrations in sediments. A similar increase in β-glucosidase activity was already reported by Paumelle et al. (2021) in leaf litter-associated microbial communities exposed for 16 days to a nominal concentration of 5 μg L^–1^ of SMX or SMZ with a difference in the magnitude of the observed response according to the presence or absence of periphytic communities. They hypothesized that the β-glucosidase increase was due to the use of an external C supply in the antibiotic treatments and/or to an adaptative response to resist antibiotics (Paumelle et al., 2021). By contrast, Kergoat et al. (2021) observed no change in the β-glucosidase activity of periphytic biofilms exposed for 28 days to SMX concentrations between 0.2 and 4 μg L^–1^. Moreover, Liu et al. (2016) observed a negative effect of SMX on the β-glucosidase activity of soil microbial communities, but the concentrations they used (5 and 50 mg SMX kg^–1^ dw soil) were far higher than the environmental concentrations tested here.

On contrary to SMX, SMZ exposure only had slight effect on β-glucosidase activity and respiration (Figures 2A,E). Thus β-glucosidase activity remained similar to controls in sediments exposed to low concentrations of SMZ whereas exposure to high concentration of SMZ led to β-glucosidase activities lower than controls after 21 days of exposure (Figure 2A). To our knowledge, this is the 1st report of β-glucosidase inhibition by exposure to environmentally relevant concentrations of SMZ. This inhibition may indicate a higher toxicity of SMZ than SMX or a higher exposure of sediment microbial communities to SMZ than SMX. Indeed, the higher concentration of SMZ than SMX indicated that microbial communities attached to sediments are likely to be more exposed to SMZ than to SMX. Nevertheless, further investigations are needed to determine whether these differences in toxicity are linked to different modes of action or to different behavior within the aquatic ecosystem.

In the present experiment, the chronic exposure to SMX also tended to stimulate the aerobic respiration of sediment communities (Figure 2E). It was thus positively correlated to SMX concentrations in sediment exposed to the high-SMX condition (*r* = 0.726, *p* < 0.05). In addition, after 28 days of exposure to the low-SMX condition, aerobic respiration was higher than in controls. Given the marked decrease in SMX concentrations (about 49%) observed in the low-SMX treatment between D21 and D28 (Figure 1A), it can be hypothesized that the increased production of CO_2_ observed at D28 could be at least partly due to the mineralization of the antibiotic. However, this hypothesis must be taken with caution, since no significant increase in aerobic respiration was observed at D28 in the high-SMZ and low-SMZ treatments (Figure 2E), although sediment SMZ concentrations significantly decreased in both treatments between D21 and D28 (Figures 1B,D).

The contamination of sediment by SMZ affected all the measured activities positively or negatively (Figure 2), but the effects were only transient and varied according to the type of activity and the antibiotic concentration, as previously reported by Kergoat et al. (2021) with periphytic biofilms. Surprisingly, the effects of the high-SMZ treatment on β-glucosidase were opposite to those observed here in the high-SMX treatment (Figure 2A) and to those previously reported by Paumelle et al. (2021) with leaf-litter microbial communities chronically exposed to SMZ. In the present study (Figure 2A), β-glucosidase activity from sediments exposed to high concentration of SMZ was about 20% lower than control levels at D21 (*p* < 0.05) and D28 (not significant). In sediments under high-SMZ exposure, the phosphatase activity (Figure 2C) was also lower than the control level, especially at D7 (about 20% lower, *p* < 0.05) and D28 (about 30% lower, *p* < 0.05). This last observation is in line with Kergoat et al. (2021), who found that the phosphatase activity of periphytic biofilms could be sensitive to chronic exposure to SMZ. Yang et al. (2020) also observed a significant inhibition of this activity in soil exposed for 28 days to SMZ concentrations ranging from about 14 to 223 mg SMZ kg^–1^ dw soil. Whereas β-glucosidase and phosphatase activities (Figures 2A,C) were not sensitive to low levels of SMZ (<4 μg SMZ kg^–1^ dw), leucine aminopeptidase activity (Figure 2B) was found to be transiently stimulated at both low and high levels of SMZ exposure after 14 days of exposure (*p* < 0.05). This transient microbial response had also been observed previously by Kergoat et al. (2021) in periphytic biofilms exposed to mean SMZ concentrations ranging from about 106 to 157 ng SMZ L^–1^. To our best knowledge, there is no other published study investigating the chronic effect of SMZ on the leucine aminopeptidase activity. However, there is evidence that this antibiotic can impact the N-cycle in different environmental compartments including soil (D’Alessio et al., 2019) and sediment (Hou et al., 2015), through changes in denitrification processes. This was also the case here (Figure 2D), where we observed a slight but significant transient inhibition of denitrification in the high-SMZ treatment at D7 (*p* < 0.05) and a significant transient stimulation of this metabolic activity in the low-SMZ treatment at D14 and D21 (*p* < 0.05).

## Conclusion

The contamination of surface water by environmental dissolved concentrations of sulfamethoxazole and sulfamethazine led to the accumulation of these two antibiotics in surface sediments. The mean maximum concentrations measured in the sediment (about 2.1 μg SMX kg^–1^ dw and 4.5 μg SMZ kg^–1^ dw) were representative of those reported in rivers contaminated by sulfonamides. The resulting chronic exposure over the 28-day experiment had contrasting effects on the measured activities of the sediment microbial communities, depending on the substance (SMX or SMZ), the exposure level and the exposure duration. The observed differences in toxicity of 2 antibiotics from the same class, the sulfonamides SMX and SMZ, should incite to be cautious when extrapolating toxicity results from one antibiotic to another from the same class. While the SMZ treatments resulted in only transient effects on the five microbial activities investigated, we observed a significant stimulation of the β-glucosidase activity over the 28 days in the communities exposed to the high concentrations of SMX. Together with the stimulation of aerobic respiration at low SMX concentrations and the reduced concentration observed in the last days, our results suggest a potential biodegradation of this sulfonamide by microbial communities from sediments. Further research is needed to determine to what extent those communities can contribute to the biodegradation of these pharmaceuticals. Given the key functional role of surface sediment microbial communities in streams and rivers, our findings suggest that the frequently reported contamination of sediments by sulfonamides is likely to affect microbial activities supporting biogeochemical cycles with a possible impact on ecosystem functioning.

## Data Availability Statement

The raw data supporting the conclusions of this article will be made available by the authors, without undue reservation.

## Author Contributions

SP, PB-H, and CB conceived and designed the study. SP, LK, and CB organized and performed the microcosm experiment. LK, LB, and CB were responsible for the microbial analyses. LP and PB-H were responsible for the chemical analyses. LB and CB conducted the statistical analyses. SP and CB drafted the first version of the manuscript. All coauthors analyzed and interpreted the data, contributed to subsequent revisions to the manuscript and approved its final submitted version.

## Conflict of Interest

The authors declare that the research was conducted in the absence of any commercial or financial relationships that could be construed as a potential conflict of interest.

## Publisher’s Note

All claims expressed in this article are solely those of the authors and do not necessarily represent those of their affiliated organizations, or those of the publisher, the editors and the reviewers. Any product that may be evaluated in this article, or claim that may be made by its manufacturer, is not guaranteed or endorsed by the publisher.
